# Effect of Waste Cigarette Butt Fibers on the Properties and CO_2_ Footprint of Bitumen

**DOI:** 10.3390/ma18092059

**Published:** 2025-04-30

**Authors:** Kai Yang, Cheng Cheng, Yong Yan, Qinglin Wu, Ru Du

**Affiliations:** 1 School of Civil Engineering, Southwest Forestry University, Kunming 650224, China; 15687200390@163.com (K.Y.); wql18188499081@163.com (Q.W.); 18183666479@163.com (R.D.); 2 Yunnan Provincial Key Laboratory of Wood Adhesives and Glued Products, Southwest Forestry University, Kunming 650224, China; 3 Faculty of Civil Engineering and Mechanics, Kunming University of Science and Technology, Kunming 650500, China; yanyong@stu.kust.edu.cn

**Keywords:** waste acetate fibers, bitumen, rheological properties, fatigue cracking, CO_2_ footprint

## Abstract

This research utilized recycled acetate fibers from discarded cigarette butts (CBs) as reinforcing materials, reducing solid waste and enhancing the properties of bitumen. The surface properties of the fibers significantly impacted the binder characteristics. The treatment of CB fibers with anhydrous ethanol was employed to remove the plasticizer glycerol triacetate (GTA), enabling the better homogeneity of the fibers in the binder. Thermogravimetric analysis (TGA) and scanning electron microscopy (SEM) were used to assess the effectiveness of the fiber treatment. A dynamic shear rheometer (DSR) was used to explore the properties of bitumen with varying CB contents (0%, 0.25%, 0.75%, and 1.25% by weight). A whole life cycle analysis further confirmed the eco-efficiency of CB binders. The results show that the pretreatment effectively removed GTA, leading to a more homogeneous dispersion of fibers in the binder. Adding CBs can significantly improve bitumen properties, but this effect does not increase with higher dosages; when the CB content exceeded 1.25%, a reduction in fatigue resistance was observed. Among the tested dosages, the optimal amount was 0.75%, which improved the high-temperature performance of the binder by 2.7 times, the medium-temperature fatigue life by 1.78 times, and the low-temperature performance by 1.08 times. In terms of ecological benefits, the addition of CB fibers to bitumen pavement reduced carbon emissions by two-thirds compared to traditional bitumen pavement, resulting in a significant decrease in carbon emissions. This study provides valuable insights into the construction of sustainable transportation infrastructure.

## 1. Introduction

According to the World Health Organization, the number of smokers around the world has gradually decreased from 2000 to 2025, but smokers remain a large group. They are expected to account for up to 1.299 billion of the world’s population in 2025, or 16.5% [1]. According to the Lancet, global cigarette consumption reached 7.4 trillion in 2019, equivalent to 20.3 billion cigarettes daily. As a result, there is a lot of tobacco waste [2]. Many discarded cigarette butts are not properly disposed of. Carcinogenic substances adsorbed on the cigarette can seep into the soil, runoff, and groundwater, eventually polluting the sea and land [3,4,5].

Among them, the primary component of the cigarette filter is acetate fiber, which is derived from cellulose through the process of acetic acid treatment and effectively filters harmful substances produced during cigarette combustion [6]. As a result, many academics have investigated this significant issue. Research has shown that the carbonization of cigarette filters can effectively adsorb heavy metals, gas molecules, and nitrosamines [7,8,9] and that the performance of this derived carbon material is better than that of commercial carbon materials. Subsequently, waste cigarette butt-derived carbon materials have been shown to have better applications in hydrogen absorption, photothermal conversion material preparation, bio-oil preparation, and batteries [10,11,12]. Adsorbed chemical components from discarded cigarette butts can be prepared as metal corrosion inhibitors, insecticides, etc. [13,14].

Bituminous pavements have a distinct advantage for use on highways due to their unique properties. However, bituminous pavements are prone to rutting, cracking, and other damage when subjected to long-term service at high temperatures and heavy loads [15], thus increasing the requirements of bituminous pavements. In recent years, fibers have been widely used in adhesives as a high-performance additive with excellent physicochemical properties, according to a wide range of scientific research [16,17]. Zhang et al. found that the addition of fibers to bitumen mortar resulted in stress redistribution and a reduction in the average stress value [18]. Tang et al.’s findings indicated that the incorporation of fibers could enhance the interface strength, with basalt fiber exhibiting a particularly strong improvement [19]. Bonica et al. studied the impact of cellulose-based fibers on the mechanical properties of bitumen mortar used in paving, demonstrating that these fibers can enhance the performance of hot mix bitumen mortar [20]. Due to their large quantities, lightweight nature, and heat dissipation properties, fibers from discarded cigarette butts have been used in construction materials. Mohajerani et al. [21] added discarded cigarette filters to clay bricks to offset the energy generated during the transport of the substrate bitumen. Abbas Mohajerani et al. [22,23] used the properties of acetate fibers, such as their porous and lightweight nature, to combine paraffin-wrapped butts with a bitumen mixture, which reduced the density of the mix and reduced the heat transfer. Md Tareq Rahman [24,25] applied waste cigarette butts as cellulose fibers in bitumen concrete, significantly improving its physical properties and rheological properties. Hu et al. found that CBs enhanced the shear resistance and high-temperature stability of the bitumen binder but reduced its low-temperature crack resistance and storage stability [26]. All of the studies described above used discarded cigarette butts.

Meanwhile, another source of concern is that during the manufacturing of cigarette filter rods in factories, the filter rod may be too long or short, the diameter may be incorrect, or the glycerol content may be unsuitable, resulting in diacetate filter rods that do not meet the use standard, resulting in a large amount of filter rod waste [27]. This category of discarded cigarette butts represents unused acetate, which is more environmentally friendly than carcinogen-containing discarded cigarette butts. According to a survey, in producing a ton of diacetate filter rods, approximately 0.5–1.0% of a production machine in regular operation’s products will be substandard. Unfortunately, this part of the waste fiber is rarely utilized.

Therefore, recycling factory waste cigarette fiber bundles as a reinforcing additive improves the performance of bitumen and provides new uses for waste cigarette fibers. However, due to the presence of plasticizers between waste cellulose acetate fibers, they cannot be uniformly dispersed in bitumen, which limits their enhancement performance. Anhydrous ethanol was used to pretreat the waste cigarette butts to eliminate the influence of GTA and scanning electron microscopy (SEM) and thermogravimetric analysis (TGA) were used to evaluate the pretreatment effect. A dynamic shear rheometer (DSR) was used to test the high-temperature performance, rheological properties, fatigue resistance, and crack resistance of fiber-modified bitumen. Additionally, a whole life cycle analysis was conducted to assess the ecological benefits of fiber–bitumen. The high carbon content of wood pulp fibers was expected to reduce carbon dioxide emissions from traditional bitumen pavement and contribute to more sustainable bitumen pavements.

## 2. Materials and Methods

**Figure 1:**
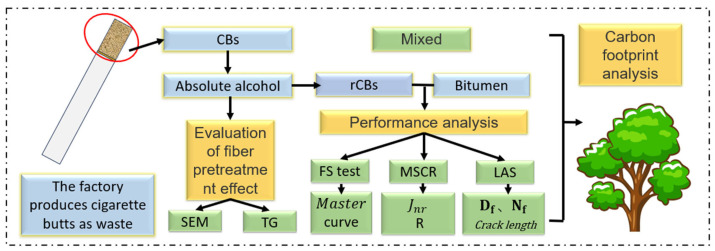
Experimental plan of the study.

The test design is illustrated in Figure 1. Experimental plan of the study. The performance of CB fiber bitumen is comprehensively assessed across three temperature ranges: high, medium, and low. Additionally, its ecological benefits are evaluated through a carbon footprint analysis.

### 2.1. Raw Materials

The bitumen used in this study was 70# raw bitumen produced by SK Group of Korea and supplied by Yunnan Bitumen Reserve and Guarantee Center of China. The technical indexes met the technical requirements of bitumen of the “Technical Specification for Highway Bituminous Pavement Construction” (JTG F40-2004). The technical indexes of the base bitumen are shown in Table 1.

### 2.2. CBs

**Figure 2:**
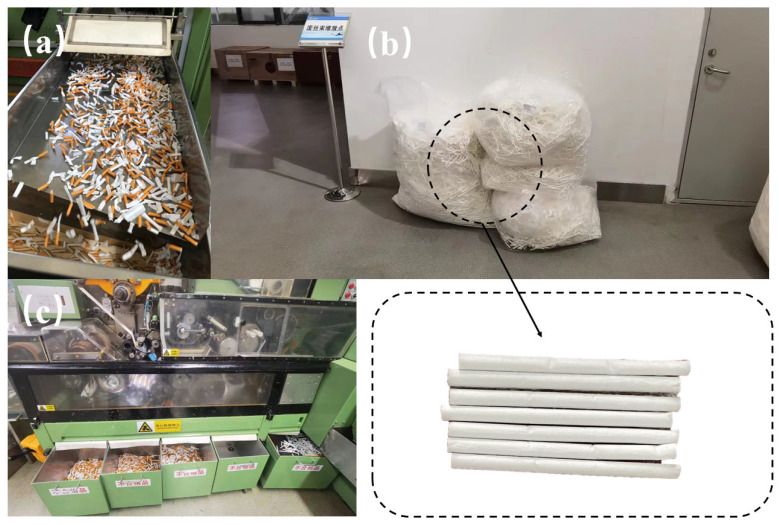
Recycling discarded cigarette butts. (**a**) Defective cigarette products; (**b**) Accumulation of non-compliant filter waste; (**c**) Non-compliant filter products.

The cigarette filters used in this work consisted of cellulose fibers with acetate, containing glyceryl triacetate and cellulose acetate, with a 6–10% mass fraction, 6–8% water, 0.2% titanium dioxide, and 1% oil agent [28]. The fiber length used was ≤6 mm, which could ensure the mechanical properties of fiber strength and deformation [29]. Yunnan Dali Tobacco Company Limited (Kunming, China) provided the fibers used in this paper, as shown in Figure 2. Among these, (b) illustrates the accumulation of non-compliant cigarette filter waste generated during filter manufacturing. Additionally, defective cigarette products from the production line further contribute to waste generation, as shown in (a) and (c).

### 2.3. Fiber Preparation and Treatment

**Figure 3:**
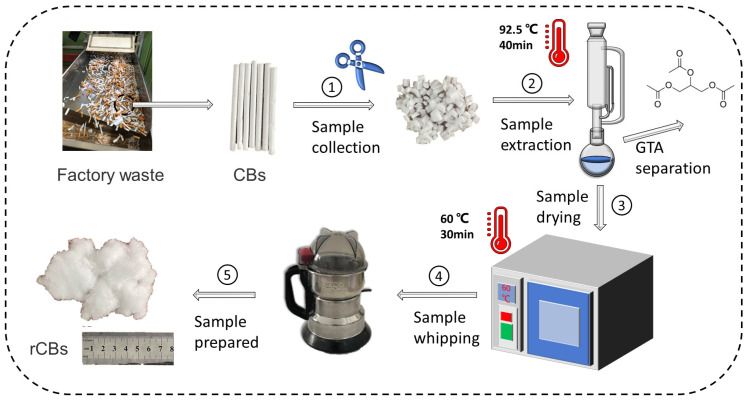
The preparation process of waste cigarette butts.

In order to increase the bonding between fibers to improve the robustness and adsorption ability of the molded filter rods, atomized glyceryl triacetate (GTA) is usually sprayed into the fibers at a specific ratio during the production process. According to gel theory, plasticizers disrupt polymer–polymer interactions via hydrogen bonding and van der Waals forces [30]. The migration of GTA increases the interactions between fibers and within molecules, making it challenging for them to flocculate and easy for them to agglomerate in the binder. Therefore, by removing the GTA, the fibers are more easily dispersed uniformly between the binders, thus enhancing performance. This paper used cigarette filters as the raw material and extracted them with a Soxhlet extractor using anhydrous ethanol as an extractant [31]. The fiber treatment process is shown in Figure 3.

### 2.4. Fiber Binders’ Preparation

In the traditional high-speed shear mixing method, the bundles of fibers lead to the fiber materials climbing the rod around the axis, leading to the fiber winding axis phenomenon in the adhesive, and forming a “fiber ball”, which is entirely unsuitable for the uniform dispersion of fibers. The divisional mixing method was thus used in this paper to solve the problem of confirming fiber dispersion.

**Figure 4:**
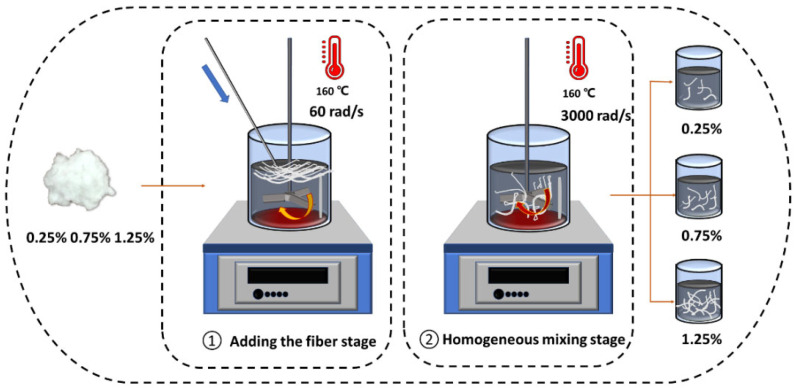
The preparation process of CB bitumen.

(1) First, 450 g of the bitumen matrix was heated in a vacuum oven at 90 °C and held for 2 h to remove moisture. The treated fibers were then added to the bitumen matrix at different percentages (0.25%, 0.75%, and 1.25% by wt. of bitumen).
(2) Using a glass rod, we pushed the loose fibers into the bitumen mastic in small quantities and in even batches.
(3) Then, we used the mixing heads at a low speed of 60 rad/s to divide the fibers in the bitumen mastic. After high-speed mixing at 165 °C, a uniform sample was made after 1 h.
(4) According to the distribution mixing method, the preparation of the acetate fiber–bitumen mixture with an acetate fiber dosing of 0.25%, 0.75%, and 1.25% was completed. This process is shown in Figure 4.

### 2.5. Evaluating the Effect of Fiber Pretreatment

The surface characteristics and morphology of CB fibers before and after pretreatment were analyzed using a TESCAN MIRA LMS type scanning electron microscope (TESCAN, Brno, Czech Republic). The fiber samples were glued to the bench using a conductive adhesive during the pre-shooting test [32] and were shot at 150–10 W. Thermogravimetric trials were applied to investigate the thermal stability of pretreated CB fibers. To comprehensively assess the effect of the pretreatment of CB fibers with anhydrous ethanol, the samples were placed in a crucible and gradually heated to 800 °C. The temperature acquisition range was between 25 and 800 °C, the heating rate was 10 °C/min, and the testing atmosphere was nitrogen. The test instrument was a Netzsch TG 209 F1 (Netzsch, Selb, Germany).

### 2.6. Frequency Sweep Test

The frequency sweep of bitumen on a dynamic shear rheometer (DSR) was performed using parallel plates of 1 mm thickness and 25 mm diameter. The frequency sweep was performed at given temperatures of 30 °C, 40 °C, 50 °C, 60 °C, 70 °C, and 80 °C in the frequency range of 0.1–100 Hz with a fixed strain of 1% according to ASTM D7175-15 [33].

### 2.7. Multiple Stress Creep Recovery Test

In order to better evaluate the high-temperature performance of bitumen, the MSCR test was conducted according to the AASHTO T350 specification [34], using parallel plates of 25 mm in diameter and 1 mm in thickness. Loading was performed for 1 s and unloading was performed for 9 s for a total of 20 creep recovery processes. The high-temperature performance of road bitumen was evaluated by recording the delayed elastic recovery deformation and irrecoverable deformation of road bitumen under external forces.

### 2.8. Damage Tolerance—Linear Amplitude Sweep

The LAS test aims to determine the resistance of bitumen binders to damage under accelerated conditions. In accordance with the AASHTO TP 101-15 specification [35], the tests were based on a dynamic shear rheometer (DSR) and were performed at 25 °C with an 8 mm oscillating plate as the mold and a spacing of 2 mm for 310 s scans. The LAS test was divided into two steps. First, the frequency scan test was performed to determine the non-damaging material parameters by applying 0.1% strain to the adhesive in the range of 0.1–30 Hz at the beginning of the test. The second step applied a shear amplitude strain to the adhesive within 0.1–30% shear strain at 10 Hz.

The aim of this paper was to consider the role of fibers in binders. The bitumen used for the tests was in its original state because cellulose does not produce new aging products after aging [36], which may have an impact on the evaluation of fiber properties for binders.

### 2.9. Crack Sprouting and Expansion

**Figure 5:**
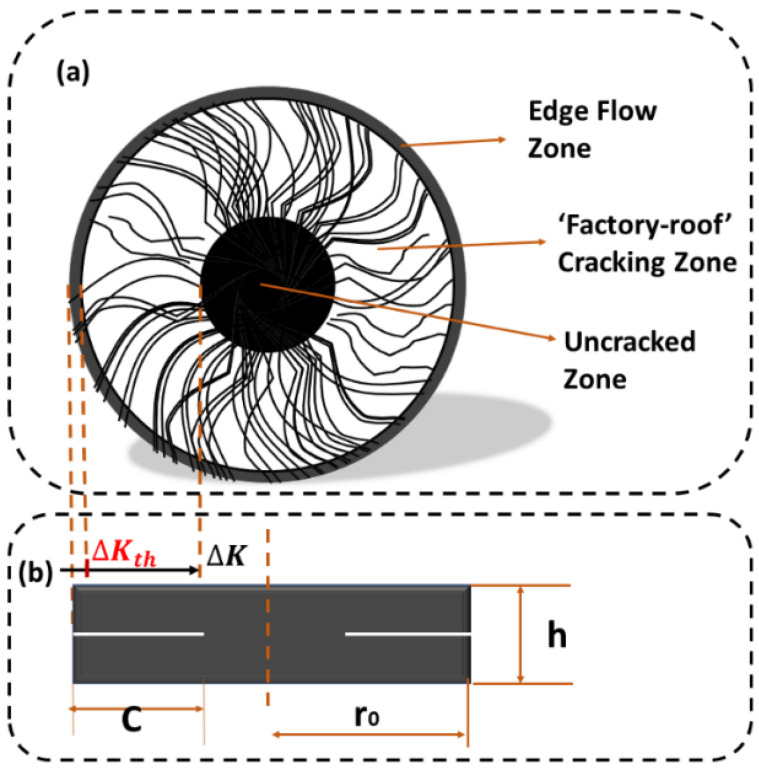
Top view of crack sprouting and expansion of CB bitumen (**a**). Front view of crack sprouting and expansion of CB bitumen (**b**).

Cracking, as one of the main forms of bitumen pavement damage [37], is a focal concern for road workers. It is recognized that the main reason for the fatigue fracture of bitumen pavement is micro-defects within the pavement under cyclic loading, micro-crack growth, and ultimately leading to the formation of macro-cracks and sample damage [38]. It is important to note that “crack initiation and growth” occur gradually in stages. In general, fatigue damage can be categorized into three stages, as shown in Figure 5.

(1) Edge flow zone: The stage where the stress intensity ∆K is less than the extended critical stress intensity ${∆K}_{th}$. Cracks sprout early in fatigue and occur in the edge region. Edge flow arises from the instability of complex edge regions [39].
(2) “Factory roof” crack zone: The stage where the stress intensity ∆K is greater than the extended critical stress intensity ${∆K}_{th}$. Under cyclic shear, the crack expands steadily toward the center, forming a rough region on the specimen surface similar to a factory roof [37].
(3) Crack-free zone: The region where cracks do not occur in the center of the specimen.

Therefore, the combination of the DSR-C model in Equation (1) can be used to express the crack expansion under rotational shear fatigue loading.

(1) $$C=r_{0}\left[1-{\left(\frac{\left|G_{n}^{*}\right|/\sin\delta_{n}}{\left|G_{0}^{*}\right|/\sin\delta_{0}}\right)}^{0.25}\right]$$

where *C* is the crack length of the binder specimen; $r_{0}$ is the initial ratio of the sample; and $\left|G_{n}^{*}\right|$ and $\delta_{n}$ are the shear modulus and phase angle of the binder at the nth damage cycle, respectively.

## 3. Results and Discussion

### 3.1. Fiber Thermal Stability Analysis

**Figure 6:**
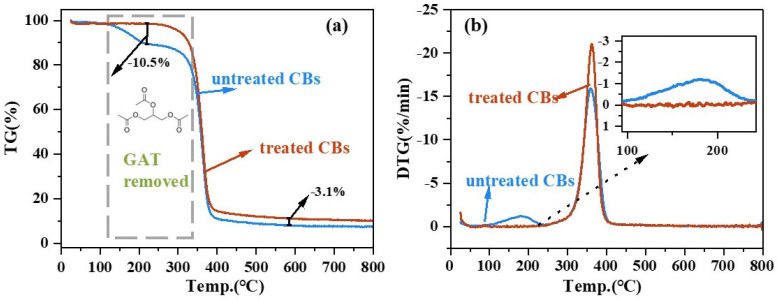
Thermogravimetric (TG) analysis chart (**a**). Pyrolysis decomposition rate (DTG) (**b**).

Figure 6 shows the thermogravimetric (TG) analysis curves and the pyrolytic decomposition rate (DTG) analysis curves for the raw and alcohol-treated cigarette filter fibers, respectively.

In Figure 6a, the changing mass of the untreated cellulose acetate (CBs) is compared with that of treated cellulose acetate (r-CBs) in the temperature interval of 128–222 °C. The heat loss rate of the CBs was 10.50%, where the lost mass was precisely equal to the mass of GTA 10% [28] in the discarded cigarette butt. Chen et al. [40] discovered that GTA was primarily removed when the temperature reached 300 °C in activated carbon adsorption of GTA, further proof that it was GTA that was released in the temperature range of 128–222 °C. In Figure 6b, no thermal decomposition of rCBs occurred between 100 °C and 200 °C, indicating that the alcohol treatment method can improve the thermal stability of cigarette filter fibers to meet the requirement of 160 °C to 200 °C in bitumen modification processing.

### 3.2. Evaluation of Fiber Pretreatment

To analyze the pretreatment degumming effect of anhydrous ethanol on CBs and the surface morphology of the fibers, the microstructure of CBs before and after surface treatment was observed by means of scanning electron microscopy.

**Figure 7:**
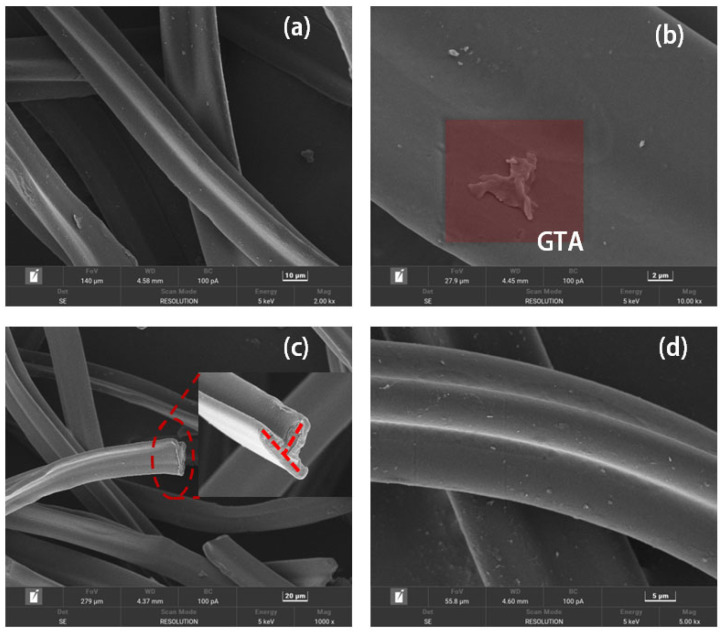
Microstructure of CBs (**a**,**b**). Microstructure of rCBs (**c**,**d**).

A Y-shaped cross-section of untreated CB fibers with smooth surfaces is shown in Figure 7a,b. The residual plasticizer GTA on the surface of CBs caused the fibers to be interwoven, making them challenging to disperse, to the detriment of the fibers, and causing them to be easily agglomerated in the binder [41]. In Figure 7c,d, the fiber surface still shows a Y-shape, with no blocky GTA on the surface, indicating that the alcohol treatment can effectively remove the plasticizer GTA. Moreover, the surface of rCBs was rougher, which increased the specific surface area of the fibers in contact with the bitumen to facilitate the embedding of fibers.

### 3.3. Complex Shear Modulus Master Curve

**Figure 8:**
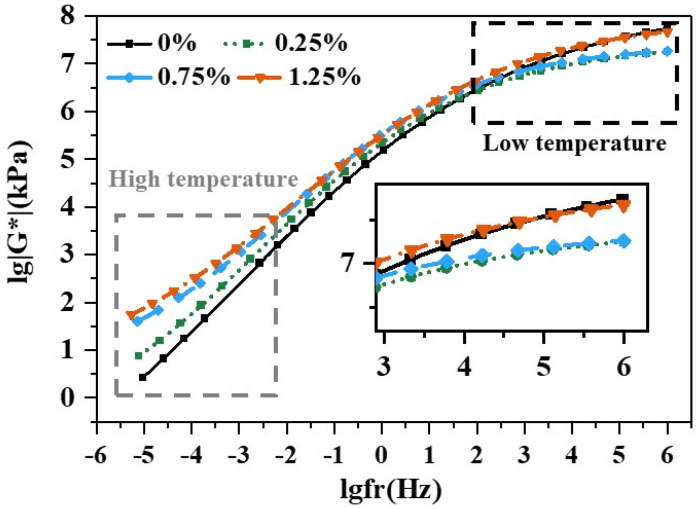
Master curve with different CB contents.

The complex shear modulus master curves at 30 °C for different CB doping levels in Figure 8 show that different CB doping levels had different effects on the binder, especially in the high-frequency (low temperature) and low-frequency (high temperature) stages, where the differences were significant. In the low-frequency phase, CB bitumen had a higher dynamic modulus than the matrix; this difference increased with the amount of admixture and reached a maximum at 1.25%. The results indicate a higher ability to resist deformation at high temperatures. From a “polymer long chain” perspective, the random distribution of fibers absorbed most of the concentrated stress and kept the bitumen flexible [42], ensuring the high-temperature stability of the adhesive.

In the high-frequency stage, the dynamic moduli of 1.25% CB bitumen and basic bitumen were almost consistent, while 0.25% and 0.75% CB bitumen had a lower dynamic modulus, respectively, with better low-temperature crack resistance. This shows that though the 1.25% CB–binder mixture was more effective at the high-temperature stage, its performance decreased with the increase in fiber doping, resulting in a hardened binder consistency [43], and the deeper hardening at low temperatures led to cracking [44]. The 0.75% CB–binder mixture was significantly better in its overall performance.

### 3.4. High-Temperature Performance

**Figure 9:**
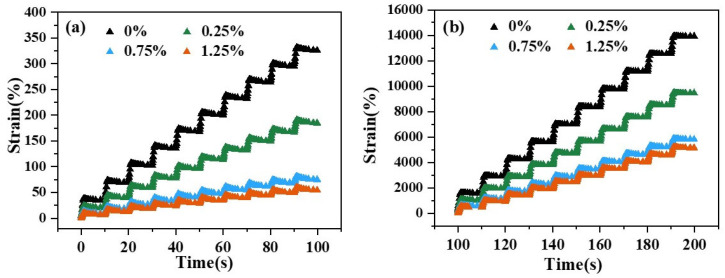
MSCR time–strain curves of binders with different CB contents at 58 °C: (**a**) 0.1 kPa; (**b**) 3.2 kPa.

To further analyze the high-temperature performance of CB fibers and binders, multi-stress repeated creep tests at 58 °C were conducted for bitumen with different CB doping levels. Figure 9 shows the curves of the time–strain response of other adhesives at 58 °C.

Bituminous pavements were more vulnerable to damage under severe loads [45], as evidenced by a greater cumulative strain value within 100 s of 3.2 kPa compared to 0.1 kPa. The results show an apparent decreasing trend in the accumulated strain value of the binder when CB fibers were added, while the binder’s high-temperature performance was significantly improved. Interestingly, strain values declined rather slowly until the fiber content rose to over 0.75%, with a nearly indiscernible reduction at 1.25% CB compared to 0.75%.

**Figure 10:**
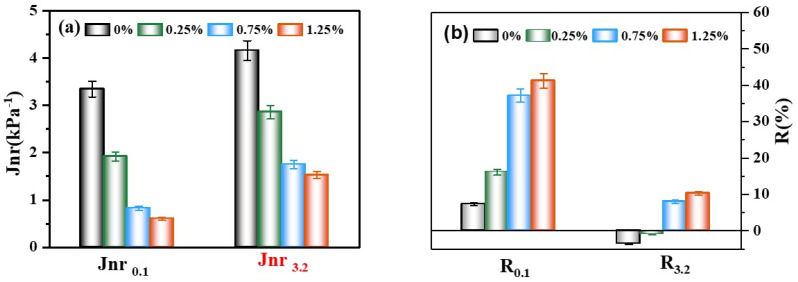
The non-recoverable creep compliance of binders with different CB contents (**a**). The creep recoverability of binders with different CB contents (**b**).

Figure 10a shows that the matrix bitumen had substantially higher irrecoverable flexibility, Jnr, than the CB bitumen at either 0.1 or 3.2 kPa stress response. As the fibers increased, the amount of Jnr was more significant; this trend may be explained by the fact that CB fibers absorbed lighter components from the binder [46], which, from the viewpoint of the colloidal hypothesis, was seen as “increasing the viscosity”. As shown in Figure 10b, the strain recovery rate of CB bitumen was significantly greater than that of the bitumen matrix, and the high-temperature performance of the mixtures with 0.75% and 1.25% CBs was significantly better than that of the bitumen matrix. However, with 1.25% fiber content, Jnr and R appeared to decrease, and high-temperature performance improved by only a little. The binder lost its elastic capacity when the bitumen matrix and 0.25% CBs had negative R_3.2_ values during excessive loading. R_3.2_ rose with admixture content, regaining viscoelastic properties. The CB fibers improved the binder’s high-energy deformation resistance, especially under heavy loads.

### 3.5. Fatigue Performance

**Figure 11:**
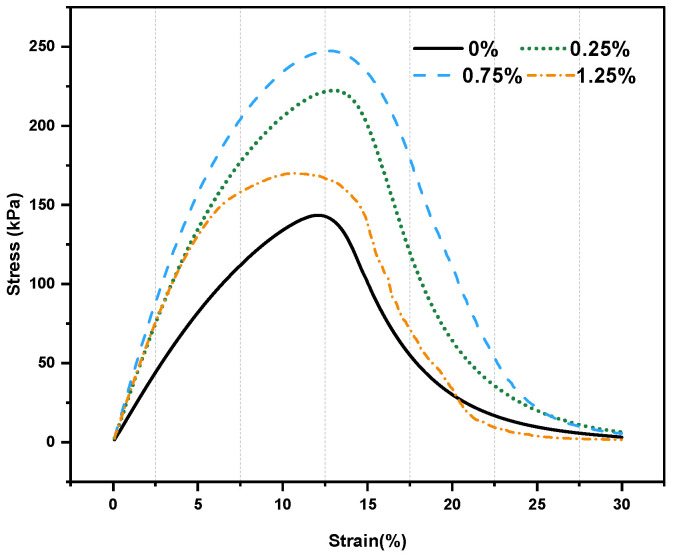
LAS-based stress–strain curves.

Based on the LAS test, the strain–stress variation curves for different adhesives are shown in Figure 11. Initially, the stress varied with strain, while after the peak strain, the stress decreased with increased strain. Due to different doping levels, the CB–binder mixtures had higher peak stress and wider strain intervals than the binder matrix. Owing to this, the fibers in the binder increased the toughness, allowing it to overcome higher stress. However, it is interesting to note that the peak strain of the binder decreased when the doping level exceeded 1.25%, being even lower than that at 0.25%, indicating that more fibers made the binder stiffer and shortened its fatigue life.

**Figure 12:**
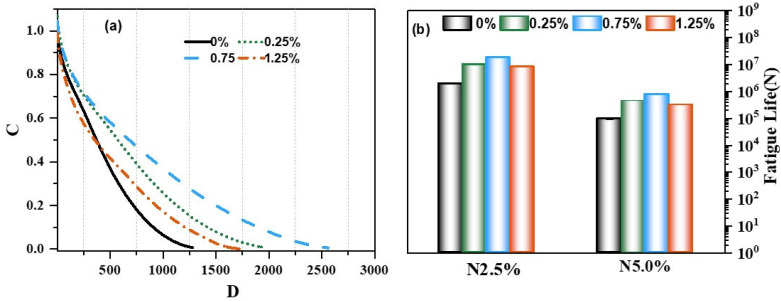
VECD-based C-D diagram (**a**); prediction of the fatigue life of bitumen (**b**).

The LAS results for predicting the lifetime of different adhesives based on the PES VECD damage model are displayed in Figure 12, demonstrating that the material integrity parameter C for the four adhesives substantially decreases with increasing cumulative damage, as expected from the fatigue damage curves (VECD) shown in Figure 12a. The integrity parameter C was obtained by normalizing the initial undamaged parameter |G*|sin at a 0.1% strain rate, and high values of C and low values of D corresponded to each other. Adding CBs to the binder effectively increased the damage resistance, as the C values of 0.75%, 0.25%, 1.25%, and 0% for the same D demonstrate; the law was the same when the material was entirely damaged (C = 0) at the same C.

In Figure 12b, the number of damage cycles *Nf* for various binders subjected to strains of 2.5% and 5.0% amplitude is shown. Strain reduced the service life of all four binders, indicating that high deformation, such as bulging and wave damage, shortens the lifespan of bitumen. The life of the binder was extended with the addition of CBs, as evidenced by the fact that the number of cycles of the CB fiber–binder mixtures at a 2.5% or 5.0% strain level was greater than that of the binder matrix. However, the issue remained the same, and as expected, the overall load-bearing capacity of the binder decreased at dosing levels above 1.25%.

### 3.6. Crack Growth Characteristics

The CB–binder mixture was found to have the ability to retard the rate of crack expansion in the specimen in the “factory-roof” area, with the base bitumen and 0.25% and 0.75% CB mixtures having comparable crack lengths by the end of the crack expansion but having a higher number of cycles compared to the base binder. For the same crack length, the higher the CB content, the more cyclic shear it could withstand, and, consequently, the greater the positive response to fatigue resistance. However, after reaching 1.25% fiber content, all aspects of the binder performance declined. The reason for this was that while the addition of fibers reduced the consistency of the bitumen and caused it to harden, appropriate fibers still made the binder behave like an elastic solid and resist fatigue cracking under repetitive traffic better than the base bitumen [47]. In contrast, excessive fibers caused the binder to harden excessively and crack more readily.

**Figure 13:**
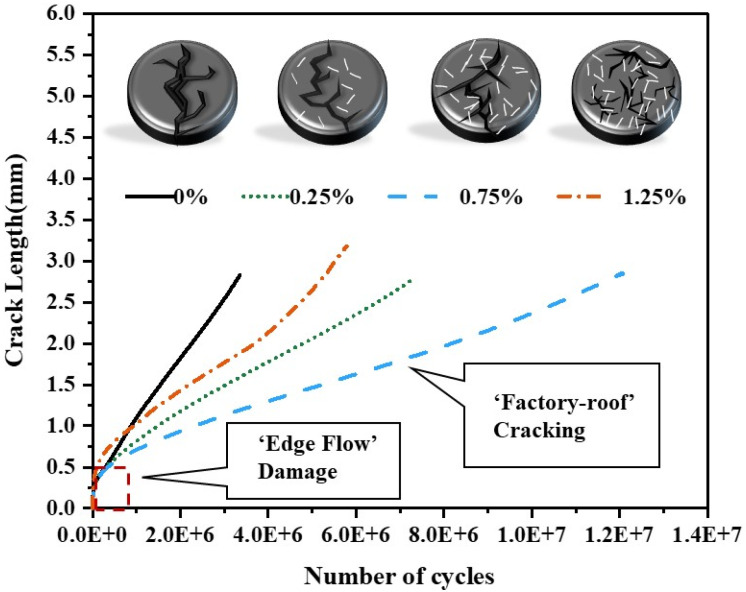
Crack length versus the number of cycles.

The phenomenon of adhesive cracking related to fiber content, as shown in Figure 13, led us to conjecture that when the matrix adhesive was stressed, the adhesive absorbed all applied stresses until it broke down. Furthermore, the adhesive unimpededly transmitted the cracking energy, resulting in a fast cracking rate and a short fatigue life. When CB fibers were added to the binder, the applied stress was carried not only by the original binder but also by the applied fibers, requiring more stress to break the fiber–binder bond, which was consistent with the LAS findings. Furthermore, the fiber strength was greater than the adhesive, resulting in a “detour” behavior of the cracking energy, which inhibited crack expansion and increased fatigue life. Nonetheless, when fiber doping exceeded 1.25%, the crack length increased. This can be explained by the fact that the presence of many fibers reduced the binder content between the fibers, making the binder part between the fibers a weak area that was easily damaged, resulting in the formation of many tiny cracks.

### 3.7. Ecological Benefit Analysis

**Figure 14:**
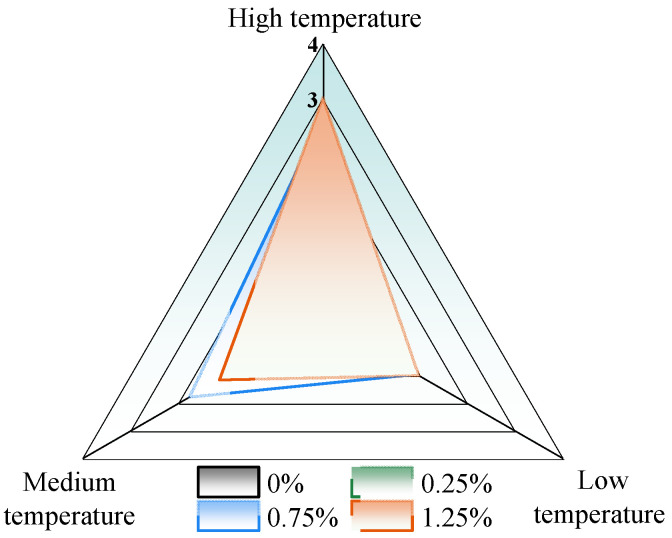
Summary of test results.

The test results can be categorized into three performance levels: high temperature, medium temperature, and low temperature. The high-temperature and low-temperature performances were derived from the master curve of the FS test, while the medium temperature performance was determined based on the fatigue life at the 5% strain level from the LAS test. All results have been normalized to the matrix bitumen, as shown in Figure 14. Among the tested dosages, the optimal amount was 0.75%, which improved the high-temperature performance of the binder by 2.7 times, enhanced the medium-temperature fatigue life by 1.78 times, and increased the low-temperature performance by 1.08 times. The ecological benefit analysis was conducted using 0.75% CB bitumen.

This paper employed the PAS2050 [48] specification to measure and analyze the life cycle carbon footprint of acetate products. Figure 14 depicts the carbon footprint of discarded cigarette butts over the entire life cycle of the acetate and its binder. We assumed that the dry weight of tree biomass in one hectare of forest land was 50–120 t (using 80 t) and that forest vegetation carbon storage was the biomass multiplied by the carbon conversion coefficient of 0.5. Given that cellulose will lose a portion of its carbon via acetate, the conversion coefficient for this paper was set at 0.4. The amount of carbon fixed in this forest equaled 80×0.4×44÷12 = 117.34 t. The amount of carbon fixed per ton of trees was calculated as 117.34 80 = 1.467 t.

**Figure 15:**
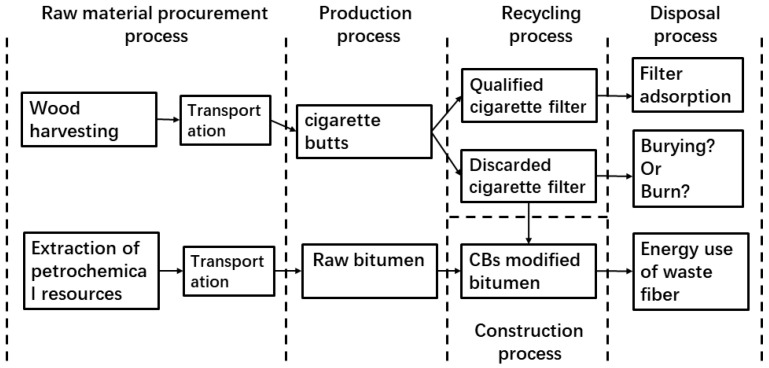
Whole life cycle diagram of acetate and adhesives.

In Figure 15, assuming 300 km between the forest land and the processing base, the trees were transported by a light truck (9 t load) with a petrol fuel consumption of 20 L/100 km and a petrol emission factor of 2.78 kg/L. The carbon footprint of transporting 1 t of trees to the processing plant was 60 2.78 = 0.1308 t. The production of 1 t of acetate would result in the storage of 1.33 t of carbon.

**Figure 16:**
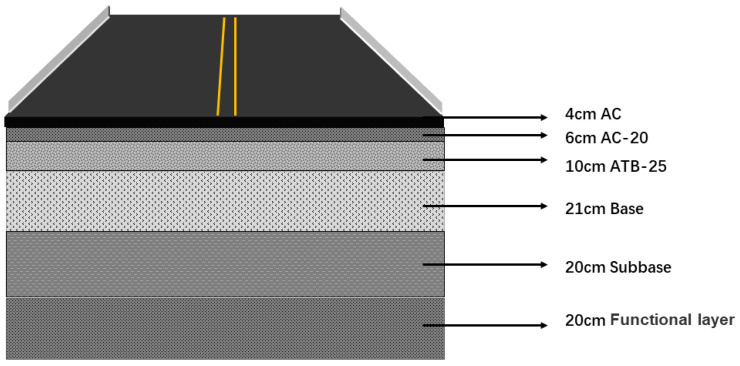
Pavement structure.

To further show the clear benefits of CB bitumen, we compared the annual carbon emissions of the total bitumen used in different highways per kilometer. Figure 16 depicts the pavement structure, assuming a design life of 15 years for the road project.

**Figure 17:**
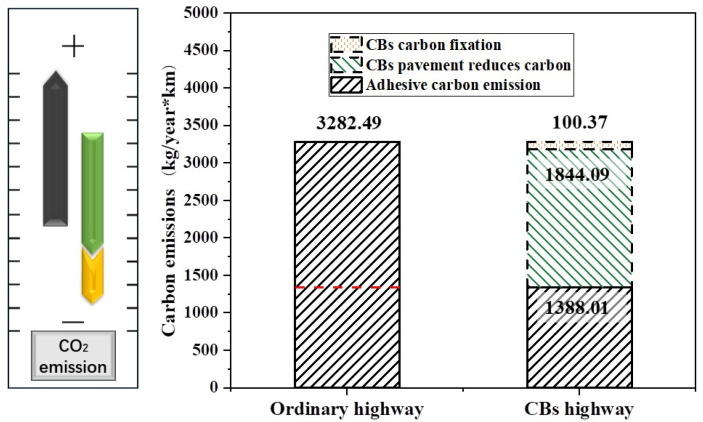
Comparison of carbon emissions from different roads. The red lines represent the carbon emissions of the CBs highway. Carbon emissions can be seen by comparison.

The annual carbon emissions per km of bitumen for the various roads were calculated from Table 2 and are shown in Figure 17. When compared to regular bitumen roads, CB–bitumen roads have significant emission reduction benefits, with the amount of carbon released from the roads reduced by two-thirds of the original amount. Using fibers as solid carbon sources, 100.37453 kg of CB fibers were permanently sequestered in the pavement. It was especially noteworthy that the carbon emission reduction achieved by improving the fatigue life of the pavement accounted for more than half of the carbon emissions of a typical road, indicating a significant effect.

Overall, the feasibility of CB fibers in practical engineering has been established. As a greener road reinforcement, they can not only effectively improve the binder’s road performance but also substitute other fiber modifiers to provide economic benefits to the project; most importantly, they can effectively reduce raw material production as well as carbon emissions in construction projects. This study therefore offers a specific reference value and ideas for the construction of green transport infrastructure.

## 4. Conclusions

Factory waste cigarette filter fibers, which are more environmentally friendly, were recycled and used as bitumen reinforcement materials to reduce waste. A series of experiments were conducted, including DSR, TGA, and SEM, to characterize the fibers before and after pretreatment, as well as the binder containing CBs. The following conclusions were drawn:

(1) Using anhydrous ethanol as an extractant completely removes the plasticizer from the CBs, making the fibers less likely to agglomerate when mixed with the binder and thus improving fiber uniformity in the bitumen. The fibers’ Y-shaped cross-section makes it easier to mix them with the binder. In addition, the binder with CBs performs significantly better than the control binder.

(2) The fibers act as a barrier in the binder, enhancing fatigue cracking resistance. When cracking energy is transferred, it takes longer to bypass the fibers, thereby extending the service life. However, when the fiber content is too high, numerous fine cracks form, which accelerates cracking and reduces cracking resistance. In conclusion, CBs have a positive effect on the binder, but an excessive amount is detrimental. The optimal dose is 0.75%, which improves the high-temperature performance of the binder by 2.7 times, the medium-temperature fatigue life by 1.78 times, and the low-temperature performance by 1.08 times.

(3) Recycled waste cigarette butts will positively impact binder modification and reduce carbon emissions during binder production, construction, and use by two-thirds compared to conventional roads. Replacing other fibers with waste materials will minimize resource waste and create an ecological–economic win–win situation.

This study primarily investigates the impact of fibers on the rheological properties of bitumen binder and evaluates the improvement of high-, medium-, and low-temperature performance with different fiber additives. Although this study yields some positive results, there are certain limitations. The scope of the research mainly focuses on the effect of fibers on the bitumen binder, without exploring the impact of fibers on bitumen mixtures. Furthermore, while we emphasize the environmental benefits of acetate fibers, we do not conduct a comprehensive life cycle analysis or explore issues such as fiber leaching or microplastics, which are crucial for a full assessment of their environmental impact.

## Figures and Tables

**Table 1:** Technical specifications of raw bitumen.

Experimental Projects	Unit	Test Result	Technology Requirements
Needle Penetration (25 °C, 5 s, 100 g)	0.1 mm	65.4	60~80
Softening Point	°C	46.6	≥46
Ductility (15 °C)	cm	>150	≥100
TFOT Needle Penetration Ratio (25 °C)	%	76.9	≥61%
Residual Ductility (15 °C)	cm	132.9	≥15

**Table 2:** Raw materials required per kilometer of pavement.

Materials	Consumption/t	Carbon Emission Factor/(kg/t)
Bitumen	260.62	189.12
CB fiber	1.954	--
Mineral powder	298.61	7.36
Cement	540.35	870.50
Aggregates	603.38	2.43

## Data Availability

The data presented in this study are available from the corresponding author upon reasonable request.
